# Decreased survival in patients treated by chemotherapy after targeted therapy compared to immunotherapy in metastatic melanoma

**DOI:** 10.1002/cam4.3760

**Published:** 2021-05-01

**Authors:** Marie‐Alix Mangin, Amélie Boespflug, Delphine Maucort Boulch, Charles‐Hervé Vacheron, Isabelle Carpentier, Luc Thomas, Stéphane Dalle

**Affiliations:** ^1^ Dermatology Unit Lyon Sud University Hospital Pierre Bénite France; ^2^ Cancer Research Center of Lyon Claude Bernard Lyon–1 University INSERM1052 CNRS 5286 Centre Leon Berard Lyon France; ^3^ Biostatistics‐Bioinformatics Department Public Health Pole Hospices Civils de Lyon Evolutive biology and biometry laboratory Université Lyon 1 CNRS UMR 5558 Villeurbanne France; ^4^ Department of Anesthesia and Resuscitation Lyon Sud University Hospital Pierre Bénite France; ^5^ Pharmacy Unit Lyon Sud University Hospital Pierre Bénite France

**Keywords:** cytotoxic chemotherapy, immunotherapy, melanoma, nivolumab, pembrolizumab, targeted therapy

## Abstract

**Background:**

Cytotoxic chemotherapy (CC) is currently used in metastatic melanoma after patients have developed resistance to immune checkpoint inhibitors (ICI) and/or Mitogen‐Activated Protein Kinase inhibitors (MAPKi). We sought to evaluate if a previous treatment by ICI or MAPKi influences clinical outcomes in patients treated by CC in metastatic melanoma.

**Methods:**

Eighty‐eight patients with a metastatic melanoma, treated by CC after a previous treatment by ICI or MAPKi between January 2009 and October 2019, were retrospectively analyzed. Progression‐Free‐Survival (PFS), Overall Survival (OS), Overall Response Rate (ORR), and Disease Control Rate (DCR) were evaluated in patients treated by CC according to their prior treatment by ICI or MAPKi.

**Results:**

Patients treated by CC after ICI tended to have a better median PFS (2.81 months (2.39–5.30) versus 2.40 months (0.91–2.75), *p* = 0.023), median OS (6.03 months (3.54–11.54) versus 4.44 months (1.54–8.59), *p* = 0.27), DCR (26.0% vs. 10.5%, *p* = 0.121) and ORR (22.0% vs. 7.9% *p* = 0.134) than those previously treated by MAPKi.

**Conclusions:**

A prior treatment by an MAPKi may be associated with a worse response to CC than ICI, and further investigations should be performed to confirm if there is a clinical benefit to propose CC in this setting.

## Graphical abstract

Cytotoxic chemotherapy may be used as a last resort after failure of a prior treatment by targeted therapy or immunotherapy in metastatic melanoma. The aim of this study was to evaluate if a prior treatment by targeted therapy or immunotherapy may influence clinical outcomes in patients treated by a second line cytotoxic chemotherapy in metastatic melanoma. We found that patients treated by cytotoxic chemotherapy after prior treatment by targeted therapy, tended to have worse clinical outcomes than patients previously treated by immunotherapy and that prior immunotherapy does not seem to potentiate response to cytotoxic chemotherapy when compared to historical cohorts.

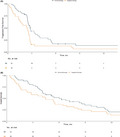

## 1 INTRODUCTION

Metastatic melanoma treatment has experienced spectacular progress in recent years with the advent of immunotherapy and targeted therapies that have replaced cytotoxic chemotherapy (CC) as first line treatments.^1^

In historical cohorts, CC used as a first line treatment showed limited clinical activity in metastatic melanoma with, overall response rates (ORR) ranging from 3 to 26% (with an average RR of 13.7%), median progression‐free survival (PFS) ranging from 1.5 to 5.6 months (with an average of 2.9 months) and median overall survival (OS) ranging from 6.6 to 15.6 months (with an average of 10.4 months).^2^

Immune checkpoint inhibitors (ICI) like ipilimumab ‐that blocks cytotoxic T‐lymphocyte antigen CTLA4 (CTLA4)‐ or nivolumab and pembrolizumab ‐that block programmed death‐ligand 1 (PD1)‐ have been approved in metastatic melanoma since 2011.^3^, ^4^, ^5^ MAPKi (mitogen‐activated protein kinases ‐MAPKi) that inhibit BRAF (vemurafenib, dabrafenib, encorafenib) have been approved first as single agents before being approved in combination with MEK inhibitors (cobimetinib, trametinib and binimetinib) in metastatic melanoma patients with *BRAF V600* mutations.^6^, ^7^, ^8^, ^9^, ^10^ These immunotherapies and targeted therapies have raised high hopes by providing improved outcomes in metastatic melanoma, and almost 50% of patients are now still alive 5 years after treatment initiation.^11^, ^12^

Unfortunately, patients who are resistant or become resistant to these therapies, have few available approved therapeutic options, and they may receive CC even though few studies have been published evaluating the clinical activity of CC in patients who have become resistant to ICI and/or MAPKi.

In vitro and in vivo data suggest that the immunomodulatory effects of CC may potentiate the effects of immunotherapy.^13^, ^14^, ^15^, ^16^, ^17^, ^18^ Temozolomide may decrease the T reg population and activation^19^ and enhance antigen‐specific T‐cell expansion during recovery from lymphopenia^20^; and dacarbazine can sensitize tumor cells to CD8+ T‐cell mediated pathways^13^ and induce local activation of natural killer cells.^21^, ^22^

Published studies in non‐small cell lung cancer (NSCLC) and in other solid cancer types suggest that a prior treatment by immunotherapy may increase response to CC.^23^, ^24^, ^25^, ^26^, ^27^, ^28^, ^29^, ^30^, ^31^ Several studies have also suggested this in melanoma, where it has been shown that a prior treatment by immunotherapy may improve response to CC by increasing the number of cytotoxic T cells in the peripheral blood in patients treated by CC, but these results have been challenged by other studies that have not reported better clinical outcomes in patients treated by CC after immunotherapy failure.^32^, ^33^, ^34^, ^35^, ^36^, ^37^

Conversely, in vitro data suggest that resistance to targeted therapies may be associated with a cross resistance to CC^38^ but few studies have evaluated if a prior treatment by BRAF and MEK inhibitors is associated with a decreased response to CC in patients.

We conducted a retrospective cohort study to evaluate if a prior treatment by immunotherapy or targeted therapy influences the response to CC in patients with a metastatic melanoma. We then compared response to CC in our cohort of patients resistant to immunotherapy and/or targeted therapy to previously published historical cohorts of patients treated by a first line CC to evaluate if a prior treatment by immunotherapy may potentiate the response to CC.

## 2 MATERIALS AND METHODS

### 2.1 Setting and participants

Inclusion criteria for this study were patients with an unresectable stage III or IV metastatic melanoma treated from January 2009 to October 2019 with CC immediately after a first line treatment by ICI or MAPKi in the Dermatology Department of the Lyon Sud University Hospital in Pierre Bénite, France. Patients who had received prior treatment by CC before ICI or MAPKi, or patients who received prior MAPKi and ICI treatment were excluded. Eligible patients were screened by interrogating the hospital pharmacy database of the Hospices Civil de Lyon and EASILY^®^, the patient records software, to identify all patients with a melanoma who were treated by CC.

The chemotherapeutic agents used in the Hospices Civil de Lyon were dacarbazine, temozolomide and fotemustine. The ICI used were pembrolizumab, nivolumab and ipilimumab. The MAPKi used were the BRAF inhibitors ‐vemurafenib, and dabrafenib, and the MEK inhibitors ‐cobimetinib, binimetinib and trametinib, that were used as single agents or in combination (vemurafenib/cobimetinib; dabrafenib/trametinib).

All therapies were continued until disease progression, intolerable toxicity, patient decision, or death.

This study was conducted in accordance with the ethical principles of the Declaration of Helsinki and consistent with Good Clinical Practice guidelines. The protocol of the study was approved by the independent ethics committee of the Hospices Civil de Lyon (approval number 19‐216), the consent of all the patients of the study still alive whose data were used was requested by sending them an information leaflet.

### 2.2 Data collection

Data collection was performed retrospectively using electronic medical records by M.A.M.

Patient characteristics included sex and age at chemotherapy initiation. Melanoma characteristics at diagnosis included American Joint Committee on Cancer stage 8 (AJCC 8th) at first systemic treatment initiation, Breslow index, melanoma mutational status, primary site, and histopathological type.

Treatment characteristics included chemotherapy drug, AJCC 8 staging at the CC initiation, number of metastatic sites prior to CC, presence, or absence of brain metastasis prior to CC, the date of the last treatment administration before CC initiation, and LDH level at chemotherapy initiation.

Data collection to evaluate disease progression included, the date of CC initiation, a prior treatment with ICI or targeted therapy, response to chemotherapy at 3 months, date of last CC treatment, reasons for stopping CC (progression, death, toxicity, patient choice), best response to CC (complete response (CR), partial response (PR), stable disease (SD), progression, not assessable), data of the best response to CC, the date of the disease progression, the date of death, and the date of the last follow‐up.

To evaluate adverse events (AEs) occurrence during CC, AEs were collected and graded according to the Common Terminology Criteria for Adverse Event (CTCAE) version 5.0.

Clinical follow‐up was scheduled every 3 or 4 weeks during treatment. Radiological follow‐up was scheduled every 3 months.

### 2.3 Outcomes

The primary endpoint was median PFS during CC treatment after a previous treatment by immunotherapy or targeted therapy. PFS was defined as the time from CC initiation to the clinical or radiological progression. Clinical progression was defined by the onset of new lesions or the increase of existing lesions and radiological progression according to the patient’s clinician.

The secondary endpoints were OS, ORR and DCR (Disease Control Rate). OS was defined as the time from the CC initiation until death due to any cause (or censoring; patients were censored at the latest date patients were known to be alive). ORR was defined as the proportion of a patient who had a partial or a total response to CC. DCR was calculated by the addition of CR, PR, and SD.

Subgroup analyses were performed in group 1 to see if the type of immunotherapy could influence the response to CC. The same endpoints (PFS, PS, ORR, and DCR) were calculated.

### 2.4 Statistical analysis

Variables were summarized by numbers and percentages for categorical data and by the median, and quartiles for quantitative data. The *χ* ^2^ or Fisher tests were used for comparisons of categorical variables. Wilcoxon tests were used to compare continuous variables for unpaired comparison. Survival analyses were performed using a parametric model. Hazard rate was estimated using cubic splines implemented in the survPen package [39, 40]. Nonadjusted and adjusted on main confounders’ hazard ratios were estimated. Response rate was analyzed with logistic regression model and odds ratios were estimated. All analyses were performed using the R software [41].

No correction was applied for multiple testing since it was considered as exploratory.

## 3 RESULTS

### 3.1 Patients characteristics

During the study period, 425 patient records were analyzed, and 88 patients who received a first line immunotherapy (group 1; *n* = 50) or targeted therapy (group 2; *n* = 38) just before CC were included.

Three hundred and thirty‐seven patients were excluded either because they received only CC (*N* = 269), first line CC before being treated by MAPKi or ICI (*N* = 40), CC after treatment by MAPKi and ICI (*N* = 25) or because they received other treatments prior to CC (*N* = 3).

**FIGURE 1:**
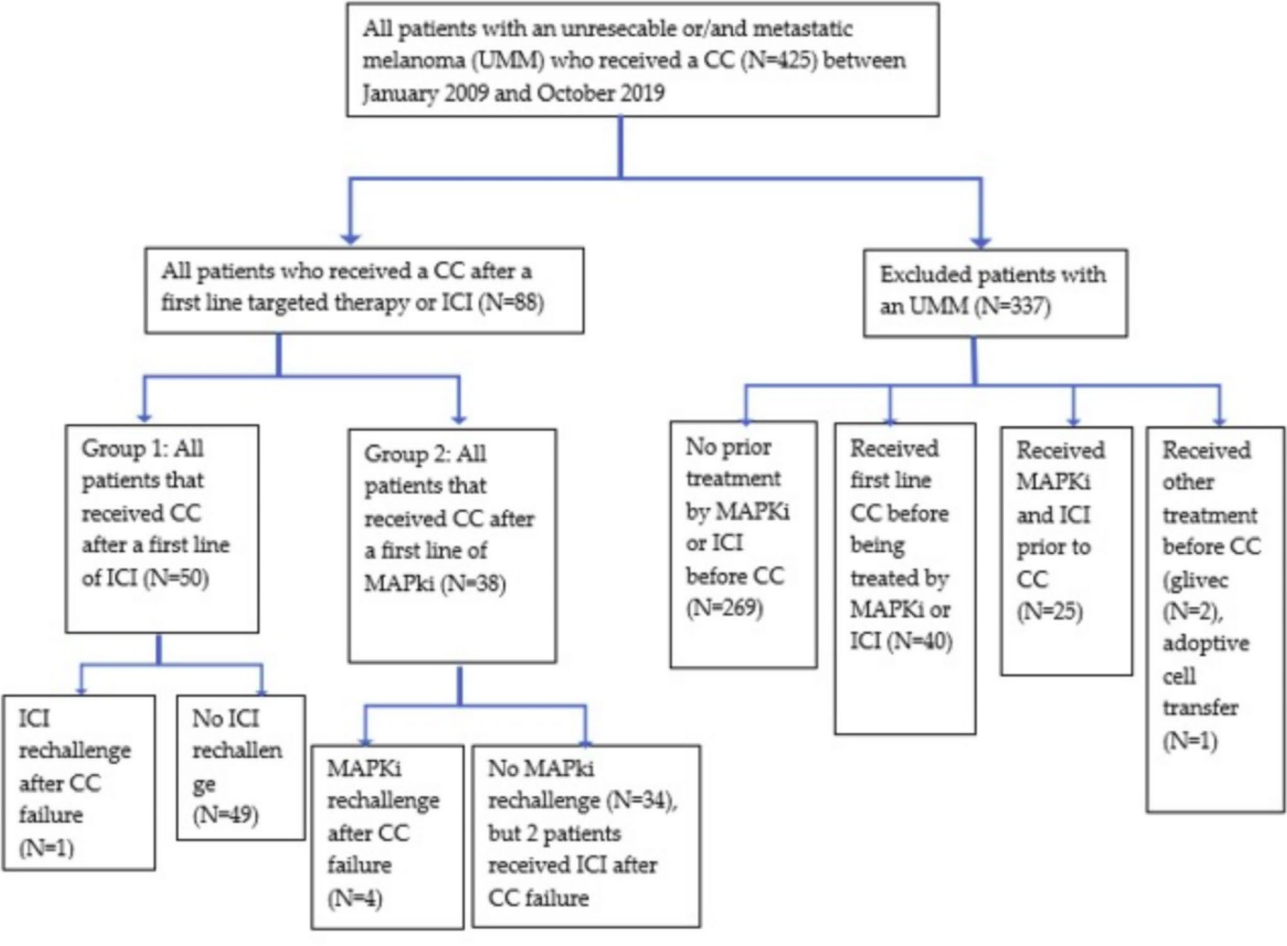
Flowchart of the study cohort. CC, Cytotoxic Chemotherapy; ICI, Immune Checkpoint Inhibitors; MAPKi, MAPK inhibitors; UMM, unresecable or/and metastatic melanoma

Among the 50 patients of group 1, one patient was rechallenged with an ICI after CC treatment; among the 38 patients of group 2, four patients were rechallenged with a MAPKi and two patients received ICI after CC (Figure 1).

Patients’ characteristics are presented in the Table 1. All reported clinical features were comparable between group 1 and 2 except concerning melanoma mutational status (68% of melanomas in patients in group 1 where BRAF, NRAS, cKIT wild type, while they were only 2.6% in group 2 (*p* < 0.001)), the type of CC (56.0% of dacarbazine in group 1 vs. 28.9% in group 2, *p* = 0.023), and the ECOG; which was significantly higher in group 1 (ECOG 3‐4 61.0% vs. 20.8%, *p* = 0.004).

**TABLE 1:** Patient characteristics in each group

	Group 1: ICI	Group 2: MAPKi	*p*‐value	Total
Demographic characteristics	*N* = 50/88	*N* = 38/88		*N* = 88/88
Age (mean (SD), years) at chemotherapy initiation (*n* = 88/88)	68.25 (13.27)	60.88 (13.71)		65.07 (13.88)
Gender (*n* = 88/88)				
Male	26 (52.0%)	25 (65.8%)		51 (58.0%)
Female	24 (48.0%)	13 (34.2%)		37 (42.0%)
Melanoma subtype (*n* = 88/88)				
ALM ‐ Acral Lentiginous Melanoma	6 (12.0%)	0 (0.0%)		6 (6.8%)
Choroidal melanoma	2 (4.0%)	0 (0.0%)		2 (2.3%)
Unclassified	3 (6.0%)	9 (23.7%)		12 (13.6%)
LMM ‐ Lentigo Maligna Melanoma	2 (4.0%)	0 (0.0%)		2 (2.3%)
Mucosal Melanoma	6 (12.0%)	0 (0.0%)		6 (6.8%)
Nodular Melanoma	4 (8.0%)	5 (13.2%)		9 (10.2%)
Unknown primitive	5 (10.0%)	1 (2.6%)		6 (6.8%)
SSM ‐Superficial Spreading Melanoma	17 (34.0%)	20 (52.6%)		37 (42.0%)
Unknown	5 (10.0%)	3 (7.9%)		8 (9.1%)
Breslow Index (mean (SD), mm) (*n* = 87/88)	3.92 (6.15)	3.30 (3.06)	*p* = 0.404 (Wilcoxon)	3.65 (5.02)
Genotype (*n* = 88/88)				
*BRAF*	0 (0.0%)	35 (92.1%)	*p* < 0.001 (Fisher)	35 (39.8%)
*NRAS*	13 (26.0%)	1 (2.6%)		14 (15.9%)
Other (GNAQ, *cKIT*)	1 (2.0%)	0 (0.0%)		1 (1.1%)
Wild type for BRAF, NRAS, GNAQ, and cKIT	34 (68.0%)	1 (2.6%)		35 (39.8%)
Unknown	2 (4.0%)	1 (2.6%)		3 (3.4%)
AJCC Stage at diagnosis (*n* = 88/88)				
I	6 (12.0%)	8 (21.1%)	*p* = 0.285 (Fisher)	14 (15.9%)
II	16 (32.0%)	16 (42.1%)		32 (36.4%)
III	19 (38.0%)	8 (21.1%)		27 (30.7%)
IV	8 (16.0%)	4 (10.5%)		12 (13.6%)
Unknown	1 (2.0%)	2 (5.3%)		3 (3.4%)
AJCC Stage at chemotherapy initiation (*n* = 88/88)				
IIIcd‐IVM1ab	7 (14.0%)	4 (10.5%)	*p* = 0.083 (Fisher)	11 (12.5%)
IVM1c	23 (46.0%)	10 (26.3%)		33 (37.5%)
IVM1d	20 (40.0%)	24 (63.2%)		44 (50.0%)
ECOG score at chemotherapy initiation (*n* = 65/88)				
0–1	16 (39.0%)	19 (79.2%)	*p* = 0.004 (Chi2)	35 (53,8%)
2‐3‐4	25 (61.0%)	5 (20,8%)		30 (46,2%)
Chemotherapy treatment (*n* = 88/88)				
Dacarbazine	28 (56.0%)	11 (28.9%)	*p* = 0.023 (Fisher)	39 (44.3%)
Fotemustine	21 (42.0%)	25 (65.8%)		46 (52.3%)
Temozolomide	1 (2.0%)	2 (5.3%)		3 (3.4%)
Number of metastatic sites prior to chemotherapy (*n* = 88/88)				
<3 or =3	29 (58.0%)	18 (47.4%)	*p* = 0.439 (Chi2)	47 (53.4%)
>3	21 (42.0%)	20 (52.6%)		41 (46.6%)
Bain Metastasis prior to chemotherapy (*n* = 88/88)				
Yes	32 (64.0%)	17 (44.7%)	*p* = 0.113 (Chi2)	49 (55.7%)
No	18 (36.0%)	21 (55.30%)		39 (44.3%)
Elevated LDH at chemotherapy initiation (*n* = 71/88)	32/42 (76.2%)	19/29 (65.5%)	*p* = 0,415 (Chi2)	51/71 (71.8%)

ICI prior to CC in group 1, were nivolumab (*N* = 15/50, 30%), pembrolizumab (*N* = 14/50, 28%), ipilimumab (*N* = 15/50, 30%), and ipilimumab+ nivolumab (*N* = 6/50, 12%).

MAPKi prior to CC in group 2 included vemurafenib (*N* = 27/38, 71.1%), dabrafenib (*N* = 4/38, 10.5%), dabrafenib + trametinib (*N* = 1/38, 2.6%), cobimetinib + vemurafenib (*N* = 3/38, 7.9%), and binimetinib (*N* = 3/38, 7.9%).

Chemotherapeutic agents used in group 1 were dacarbazine (*N* = 28/50, 56%), temozolomide (*N* = 21/50, 42.0%) and fotemustine (*N* = 1/50, 2.0%).

Chemotherapeutic agents used in group 2 were dacarbazine (*N* = 11/38, 28.5%), temozolomide (*N* = 25/38, 65.8%) and fotemustine (*N* = 2/38, 3%).

Median duration of CC was 1.49 months (0.70–2.25) in group 1 and 0.48 months (0.24–0.95) in the group 2. Median time from immunotherapy cessation to CC initiation was 2.16 months (0.85–3.16) in group 1 and median time from targeted therapy to CC initiation was 1.16 months (0.46–2.29).

### 3.2 Clinical outcomes

**FIGURE 2:**
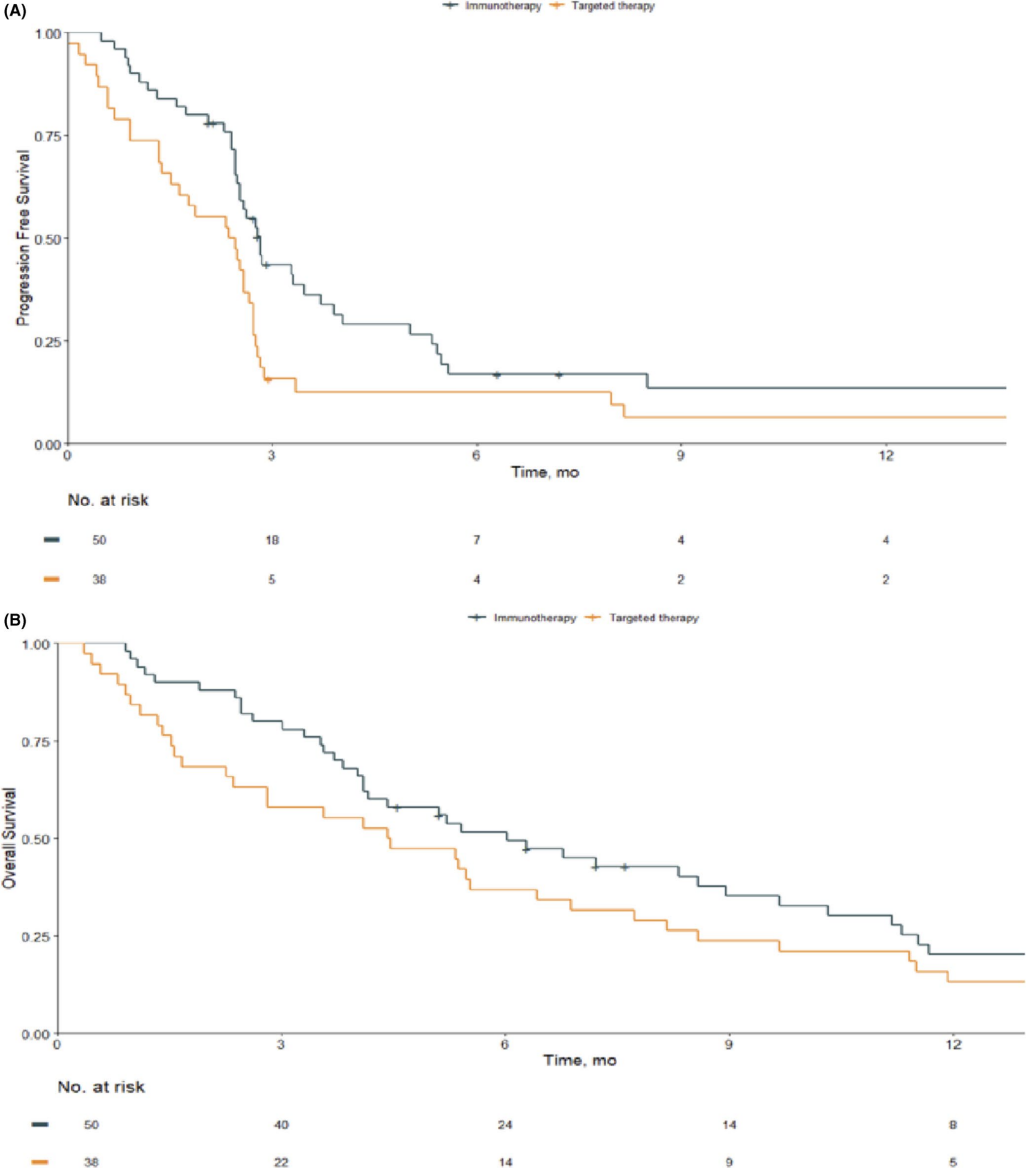
Progression‐Free Survival (A) and Overall Survival (B) to chemotherapy in function prior treatment by immunotherapy or MAPKi. This figure shows the Kaplan‐Meier Progression‐Free Survival and Overall Survival curves of all patients included in the study in function of their prior treatment before CC. (A) logrank test: 0.023; (B)logrank test: 0.27

Patients treated by ICI prior to CC (group 1) had a significantly longer median PFS ( 2.81 months (2.39–5.30) versus 2.40 months (0.91–2.75), *p* = 0.023), and tended to have a longer median OS (6.03 months (3.54–11.54) versus 4.44 months (1.54‐8.59), *p* = 0.27), a higher DCR (26% vs. 10.5%, *p* = 0.121) and a higher ORR (22% vs. 7.9%, *p* = 0.134) compared to patients treated by MAPKi prior to CC (group 2) **(**Figure 2). PFS at 6 months (PFS6) was 16.9% (8.7–32.9) in group 1 and 12.6% (5.4–29.7) in group 2, and PFS at 12 months was 13.5% (6.1–30.0) in group 1 and 6.3% (1.7–23.2) in group 2. OS at 6 months (OS6) was 51.6% (39.4–67.6) in group 1 and 36.8% (24.3–55.9) in group 2. OS at 12 months was 20.1% (11.0–66.7%) in group 1 and 13.2% (5.8–29.8%) in group 2.

Multivariate analysis, considering Breslow thickness, brain metastasis, number of metastatic sites at CC initiation, and AJCC staging at CC initiation showed no change in adjusted HRs for the observed trend for the two groups concerning PFS and OS (using the group immunotherapy as the reference group: PFS aHR = 1.61 (0.99–2.63), *p* = 0.05, OS aHR = 1.15 (0.49–2.71), *p* = 0.55, DCR aOR = 0.34).

In subgroup analysis in group 1, a prior treatment by the association of anti‐CTLA4 and anti‐PD1 seems to have improved outcomes under CC than a prior treatment by anti‐PD1 or anti‐CTLA4 alone (Table 2). However, these analyses were exploratory as these subgroups were exceedingly small.

**TABLE 2:** Overall response rate, disease control rate, overall survival and progression‐free survival under chemotherapy according to the type of IT prior chemotherapy: anti‐CTLA4, anti‐PD1 or association

Type of immunotherapy prior to CC	Anti‐CTLA4 *N* = 15/50	Anti‐PD1 *N* = 29/50	Association *N* = 6/50	Total *N* = 50
Overall response rate (*N*, %)	4 (26.7%)	3 (10.3%)	4 (66.7%)	11 (22.0%)
Disease control rate (*N*, %)	4 (26.7%)	5 (17.2%)	4 (66.7%)	13 (26.0%)
Median overall survival (months)	6.90 (2.40–11.34)	4.10 (3.16–5.72)	9.05 (6.66–12.57)	4.43 (3.02–8.95)
Median progression‐free survival (months)	2.61(1.21–4.58)	2.52 (2.34–3.07)	4.66 (3.56–6.18)	2.60 (2.06–3.70)
Death (*N*, %)	14 (93.3%)	23 (79.3%)	4 (66.7%)	41 (82.0%)

CC treatment‐related AEs of any grade were reported in 28 patients of group 1 (56.0%) and in 10 patients of group 2 (26.3%) (*p* = 0.01). Grade 3, 4 toxicities according to the CTCAE occurred in 11 patients of the group 1 (22.0%) and in three patients of group 2 (7.9%) (*p* = 0.134). The most frequent toxicities were asthenia, cytopenias, nausea, vomiting, and abnormalities of liver functions. Discontinuation of treatment due to treatment‐related AEs were noted in four (8%) patients in group 1 and in one patient in group 3 (2.6%). There were no treatment deaths reported in both groups (Table 3).

**TABLE 3:** Adverse events during chemotherapy

	Group 1: ICI (*N* = 50/88)	Group 2: MAPKi (*N* = 38/88)	*p*‐value	Total (*N* = 88/88)
Toxicities (*n* = 88/88)				
No	22 (44.0%)	28 (73.7%)	*p* = 0.01 (Chi2)	50 (56.8%)
Yes	28 (56.0%)	10 (26.3%)		38 (43.2%)
Grades 3–4 toxicities (*n* = 88/88)				
No	39 (78.0%)	35 (92.1%)	*p* = 0.134 (Chi2)	74 (84.1%)
Yes	11 (22.0%)	3 (7.9%)		14 (15.9%)
Anemia	6 (12%)	1 (2.6%)		7 (8%)
Thrombopenia	4 (8%)	2 (5.3%)		6 (12%)
Cytolysis	1 (2%)	0 (0%)		1 (1.1%)
Pancytopenia	10 (20%)	4 (10.5%)		14 (15.9%)
Neutropenia	3 (6%)	1 (2.6%)		4 (4.5%)
Asthenia	3 (6%)	1 (2.6%)		4 (4.5%)
Nausea/Vomiting	1 (2%)	0 (0%)		1 (1.1%)
Diarrhea	0 (0%)	1 (2.6%)		1 (1.1%)
Discontinuation of treatment due to treatment‐related AEs	4 (8%)	3 (2.6%)		7 (8%)

## 4 DISCUSSION

In our retrospective case study, a prior treatment by an ICI does not seem to be associated with a better response to CC in metastatic melanoma (median PFS = 2.81 months, median OS = 6.03, ORR = 22.0%) when compared to historical series of patients receiving a first line CC.^42^

Trials evaluating first line temozolomide and fotemustine showed similar responses.^43^, ^44^

Our results are in accordance with articles published by Karachaliou et al,^45^ Goldinger et al^46^ Weber et al^47^ and Ribas et al^5^ but differ from those by Hadash et al , St Jean et al and Markovi et al^32^, ^33^, ^37^ whose studies showed improved outcomes for patients treated by CC after ICI than patients treated by CC who were not previously treated by ICI. Additionally a retrospective study also suggested an improved clinical outcome in seven patients treated by carboplatin and paclitaxel after progression on ICI^36^ which suggests that the synergistic effect of ICI may be dependent of the type of CC used.

Finally, in our cohort, if a prior immunotherapy did not seem to increase the response to CC it was associated with an increased toxicity to CC that would need to be further investigated.

A previous treatment by targeted therapy was associated in our study with a pejorative response to CC compared to a prior treatment by immunotherapy (median PFS (2.81 months (2.39–5.30) vs. 2.40 months (0.91–2.75), *p* = 0.023), median OS (6.03 months (3.54–11.54) vs. 4.44 months (1.54–8.59), *p* = 0.27), DCR (26.0% vs. 10.5%, *p* = 0.121) and ORR (22.0% vs. 7.9% *p* = 0.134)).

Because of these poor clinical outcomes, treatment by CC after targeted therapy should be prospectively evaluated in comparison with other treatment options like MAPKi treatment beyond progression (TBP) ^48^; MAPKi discontinuation and rechallenge in tumors that are drug dependent ^49^; subsequent immunotherapy^50^ ; inclusion in a clinical trial ^51^ or best supportive care. ^52^, ^53^ MAPKi TBP could be proposed alone or in combination either with surgery in patients with an oligoprogression ^54^ (NCT03514901), or with a treatment that may decrease MAPKi resistance like an autophagy inhibitor (NCT03754179) or a histone deacetylase inhibitor (HDACi) (NCT02836548).

Interestingly 7.9% of patients were long responders to CC and had not progressed after 12 months of CC treatment.

The heterogeneity of the responses to CC after immunotherapy and targeted may be explained by the presence of genetic alterations, such as *ATM* mutations, which should confer chemo‐sensitivity that was not evaluated in our study.^34^ Further studies on larger cohorts of long responders to CC with next generation sequencing should be performed to better understand genetic and clinical factors that may be associated with long responses to CC in melanoma.

### 4.1 Limitations

This retrospective cohort study has several strengths and limitations.

Both groups were statistically comparable for most of the clinical features that were collected. If certain characteristics differed between each group (overexpression of BRAF mutated melanoma in group 2 and overexpression of mucosal melanoma in group 1) most clinical characteristics associated with a pejorative response to CC (presence or absence of a brain metastasis, AJCC staging, number of metastasis, and LDH level at chemotherapy initiation…) were similar in both groups.^55^

Our real‐life setting may be more representative of current practice compared to highly selected patients in clinical trials. But to compare more homogenous populations we included patients who only received a single line of treatment prior to CC which excluded patients who had received both treatments or other prior treatment. However, our two populations are heterogenous on certain characteristics. The chemotherapeutic agents, the ICI molecules and the MAPKi regimens used in each group were heterogenous, and we cannot exclude that this heterogeneity may have impacted our results in this retrospective cohort. Moreover, the ECOG stage was significantly higher in the group treated by ICI before CT than in the group treated by MAPKi before chemotherapy. Patients treated with ICI may therefore have been in a worse general condition than the patients treated by a MAPKi, which reinforces the trend observed in our study. However, these data must be interpreted with caution since there were certain missing data for the ECOG (28.4% of missing data).

Our cohort has a relatively small sample size, particularly in the subgroup analysis and the results obtained are not or weakly significant and may not be found in a larger cohort. However, it is to our knowledge, the first study evaluating if response to CC is differently influenced by a previous treatment by ICI or MAPKi in metastatic melanoma.

## 5 CONCLUSIONS

We confirm, in a real‐life setting, previously published data that prior treatment by an ICI does not seem to be associated with a better response to CC in metastatic melanoma when compared to historical series, but it is associated with a better response to CC than prior treatment by MAPKi.

Further investigations should be performed to confirm if there is a clinical benefit to propose CC after progression to MAPKi compared to palliative care or treatment beyond progression when patients cannot be included in a clinical trial.

## ETHICS COMMITTEE APPROVAL

Comité d’éthique du CHU de Lyon Est, April 24th 2020.

## CONFLICTS OF INTEREST

SD is a principal investigator in clinical trials sponsored by BMS, MSD, Novartis, Amgen, and Roche. SD received institutional research grants from BMS, Roche and MSD. The other authors declare no conflict of interest in relation to this article.

## AUTHOR CONTRIBUTIONS

Conceptualization: Stéphane DALLE, Amélie BOESPFLUG and Marie‐Alix MANGIN. Methodology: Stéphane DALLE and Amélie BOESPFLUG Formal analysis: Delphine MAUCORT BOULCH and Charles‐Hervé VACHERON Acquisition of data: Marie‐Alix MANGIN Resources: Isabelle CARPENTIER Writing—original draft preparation: Stéphane DALLE, Amélie BOESPFLUG and Marie‐Alix MANGIN Supervision, critical revision of the manuscript and important intellectual input: Stéphane DALLE and Luc THOMAS.

## Data Availability

I confirm that I have included a citation for available data in my references section, unless my article type is exempt. All data presented are available upon request.
